# 

**Published:** 1898-12

**Authors:** 


					﻿Southern Medical Record
A Monthly Journal of Medicine and^ifgcry;^r
Vol. XXVIII. ATLANTA, GA., DECBMEfc^ci^agahha ^EBACS aaaa	NO. 12.
BERNARD WOLFF, M.lD., EDid&}|,*;
Associate Editors :
A. W. GRIGGS, M. D.
j. McFadden gaston, Jr., m. d.
j. McFadden gaston, m. d.
WULLIS F. WKSTMORELANn MJn
B. M. ZETTLER, Business Managkr.
Entered at the Post-office at Atlanta, Ga., as SeCond-class Matter.
				

